# Early and Late Transcriptional Changes in Blood, Neural, and Colon Tissues in Rat Models of Stress-Induced and Comorbid Pain Hypersensitivity Reveal Regulatory Roles in Neurological Disease

**DOI:** 10.3389/fpain.2022.886042

**Published:** 2022-05-17

**Authors:** Evelina Mocci, Taichi Goto, Jie Chen, Seth Ament, Richard J. Traub, Susan G. Dorsey

**Affiliations:** ^1^Department of Pain and Translational Symptom Science, University of Maryland School of Nursing, University of Maryland Baltimore, Baltimore, MD, United States; ^2^Institute for Genome Sciences, University of Maryland School of Medicine, University of Maryland Baltimore, Baltimore, MD, United States; ^3^Department of Neural and Pain Sciences, University of Maryland School of Dentistry, University of Maryland Baltimore, Baltimore, MD, United States; ^4^Center to Advance Chronic Pain Research, University of Maryland Baltimore, Baltimore, MD, United States

**Keywords:** chronic overlapping pain conditions, stress, spinal, colon, DRG, blood, RNA seq, WGCNA

## Abstract

**Background:**

Irritable bowel syndrome (IBS) and temporomandibular disorder (TMD) are two chronic pain conditions that frequently overlap in the same individual, more commonly in women. Stress is a significant risk factor, exacerbating or triggering one or both conditions. However, the mechanisms underlying IBS–TMD co-morbidity are mostly unknown.

**Aim:**

To detect both specific and common stress-induced visceral hypersensitivity (SIH) and comorbid TMD–IBS pain hypersensitivity (CPH) genetic signatures over time.

**Method:**

Twenty-four female rats were randomly assigned to one of three experimental groups: naïve, SIH, and CPH (orofacial pain plus stress). RNA was extracted from blood, colon, spinal cord, and dorsal root ganglion 1 or 7 weeks after the stress paradigm. We combined differential gene expression and co-expression network analyses to define both SIH and CPH expression profiles across tissues and time.

**Results:**

The transcriptomic profile in blood and colon showed increased expression of genes enriched in inflammatory and neurological biological processes in CPH compared to SIH rats, both at 1 and 7 weeks after stress. In lumbosacral spinal tissue, both SIH and CPH rats compared to naïve revealed decreased expression of genes related to synaptic activity and increased expression of genes enriched in “angiogenesis,” “Neurotrophin,” and “PI3K-Akt” pathways. Compared to SIH, CPH rats showed increased expression of angiogenesis-related genes 1 week after exposure to stress, while 7 weeks post-stress the expression of these genes was higher in SIH rats. In dorsal root ganglia (DRG), CPH rats showed decreased expression of immune response genes at week 1 and inhibition of nerve myelination genes at 7 weeks compared to naïve. For all tissues, we observed higher expression of genes involved in ATP production in SIH compared to CPH at 1 week and this was reversed 7 weeks after the induction of stress.

**Conclusion:**

Our study highlights an increased inflammatory response in CPH compared to SIH rats in the blood and colon. DRG and spinal transcriptomic profiles of both CPH and SIH rats showed inhibition of synaptic activity along with activation of angiogenesis. Targeting these biological processes may lead to a more profound understanding of the mechanisms underlying IBS–TMD comorbidities and new diagnostic and therapeutic strategies.

## Introduction

Stress is a major risk factor for chronic pain conditions including chronic abdominal pain (1–5). An experimental study in humans showed psychological stress enhanced pain sensitivity (1). Stress-induced visceral hypersensitivity (SIH) is a chief complaint of irritable bowel syndrome (IBS) and animal models have demonstrated that stress increases visceral sensitivity (6–8). Activation of the hypothalamic–pituitary–adrenal (HPA) and sympathetic-adreno-medullary axes induced by stress increases the release of inflammatory mediators such as serotonin and pro-inflammatory cytokines in the colon, which contribute to the altered intestinal sensation and visceral hypersensitivity (9, 10).

Temporomandibular disorder (TMD) is another chronic pain condition and is one of the major comorbidities of IBS (11, 12). To shed light on the mechanisms underlying the co-existence of these two pain conditions we recently developed an animal model of comorbid visceral hypersensitivity (CPH), that reproduces pain in patients with TMD and IBS (13–15).

We previously reported that visceral hypersensitivity lasted considerably longer in CPH (> 13 weeks) compared to stress-induced visceral hypersensitivity (SIH) rats (3–4 weeks); however, during the first few weeks following stress the magnitude of pain hypersensitivity and its associated peripheral mechanisms, including corticotrophin-releasing factor signaling and mast cell activation were similar in SIH and CPH rats (15). In addition, we compared the response of primary afferents and dorsal horn neurons during the visceral hypersensitivity in CPH and SIH rats. Surprisingly, while peripheral sensitization persisted for at least 7 weeks in both SIH and CPH rats, different phenotypes of dorsal horn neurons were sensitized at 7 weeks in CPH rats compared to those observed at 4 weeks or in SIH rats (13). This suggests a mismatch between the condition-dependent behavior and peripheral and spinal mechanisms that contribute to visceral pain hypersensitivity.

In the present study, we characterized the transcriptomic profile of CPH and SIH rats by comparing them to naïve rats and each other. This analysis was carried out in the blood, colon, L6-S1 dorsal root ganglia (DRG), and L6-S1 spinal cord dorsal horn. For each tissue, we tested for differential expression (DE), both at the gene and gene-set level, across conditions and different time points from the exposure to stress. Next, we performed enrichment analyses to identify biological processes and pathways that were characteristic of CPH. In addition, we investigated the presence of clinically relevant biomarkers, by looking for common differentially expressed genes and gene-sets between blood and the other tissues.

## Materials and Methods

Experiments were performed on cycling adult female Sprague–Dawley (SD) rats (Envigo, Indianapolis, USA; 10 weeks old on arrival at the University of Maryland School of Dentistry animal facility). Rats were acclimated to the housing facility at least 7 days prior to entering the study. Rats were not tested for the estrous cycle stage since the excessive stress could alter the results due to prolonged daily handling for 7 weeks. All protocols were approved by the University of Maryland Baltimore Institutional Animal Care and Use Committee and conform to the guidelines for use of laboratory animals by the International Association for the Study of Pain. This study focused on intact female rats as we have shown that the currently used stress paradigm resulted in significantly shorter duration visceral hypersensitivity in both the stress and comorbid pain models in male SD rats [(7), unpublished observations]. Rats were randomly assigned to one of three experimental groups: naïve (*n* = 7), SIH (stress alone, *n* = 9), or comorbid pain hypersensitivity (CPH; orofacial pain plus stress, *n* = 8). Two additional rats were excluded since a full tissue set was not collected.

### Restraint as the Stressor for SIH and CPH Rats

Rats were restrained in Broome style rodent restrainers (4.8 cm diameter, 20 cm length) preventing movement for 2 h per day for 4 consecutive days (7, 16, 17). During the 2 h, rats were tilted at a 45° angle head up or head down in 15 min blocks alternating with 15 min blocks in the horizontal position (15). The day after the last restraint session was designated Day 1.

### Masseter Muscle Inflammation

One day prior to starting the stress protocol, CPH rats were briefly sedated with isoflurane, and Complete Freund's Adjuvant (CFA) (SigmaAldrich, F5881; 150 ml per side, 1:1 in saline) was injected bilaterally into the masseter muscles. This protocol (CFA+stress) produces the comorbid pain condition. We previously reported that saline injection into the masseter muscle followed by stress was similar to stress alone and CFA injection without stress did not induce visceral hypersensitivity (10, 14, 15).

### RNA Processing, Library Preparation, and RNA Sequencing

At 1 or 7 weeks following stress, rats were deeply anesthetized with isoflurane, decapitated and tissue (blood, spinal cord, L6-S1 DRG, and distal colon) were harvested, flash frozen on dry ice, dissected as needed, and then homogenized in Trizol according to manufacturer's protocol. Biospecimens were stored homogenized in Trizol until RNA extraction procedures.

RNA extraction, quality checks, and quantitation were performed at the Translational Genomic Lab in the School of Medicine. Library preparation and sequencing were performed by the Genomic Research Center, part of the Institute of Genome Sciences at the University of Maryland School of Medicine. RNA was extracted from four types of tissue: blood, distal colon, L6-S1 spinal cord, and L6-S1 dorsal root ganglia (DRG) at 1 week (week 1) or 7 weeks (week 7) after the induction of stress.

Libraries were prepared with NEB Ultra II Directional Library Prep kits. Samples at week 1 were sequenced on Illumina HiSeq 4000 platform using a 150 bp paired-end run (2 × 150), while samples at week 7 were run on Illumina NovaSeq 6000 using a 150 bp paired-end run as well. We used FastQC (18) and Trimmomatic (19) tools for evaluating the quality of the sequences, next we used HISAT2 (20) software to align the sequences to the reference Rattus norvegicus (Norway rat) genome assembly Rnor_6.0 (rn6) from Rat Genome Sequencing Consortium [GCA_000001895.4 GCF_000001895.5]. Finally, gene expression was estimated using HTSeq (21) which considers a gene as the union of all exons regardless of the isoform and excludes reads that overlap multiple genes.

### Differential Gene Expression Analysis

The difference in Illumina platforms used to sequence RNA samples at time week 1 and week 7 represents a source of variability in the distribution of gene expression data, determining a decrease of statistical power in the downstream differential expression analyses. To address the batch effect, we used the software ComBat-seq (22) which is an extension of ComBat (23), one of the most used tools for batch effect correction. In contrast with ComBat (23), which models gene expression data through a Gaussian distribution, ComBat-seq (22) preserves the integer nature of gene expression data by using a negative binomial distribution. We applied the batch effect adjustment separately for each tissue. The analysis with ComBat-Seq (22) proved successful in reducing the variability in the expression data explained by batch and therefore batch-adjusted counts were then analyzed using Limma-voom software (24). The voom function computes the mean-variance relationship in the count data and then uses this estimate as a weight in the linear regression model. Limma-voom (24) first transforms the counts into the logarithm of count per million (log2 CPM) as this conversion decreases the mean-variance trend and then fits a linear model for each gene and estimates the residuals. The distance between the fitted curve and the square root of the residuals is used as weights into Limma along with the log2 CPMs. We assessed differential expressed genes (DEGs) separately for each tissue and time point between the conditions SIH and CPH vs. naïve; next, we compared CPH and SIH conditions. Genes were considered differentially expressed if their adjusted *P*-value, estimated using the Benjamini–Hochberg test, was equal to or lower than 0.05. Furthermore, we prioritized the DEGs whose log2 fold change absolute value was higher than 1.

In parallel, we explored gene expression data using the weighted gene co-expression network analysis (WGCNA) whose algorithm clusters genes with similar expression profiles into modules (25). Unlike DEG analysis, this approach does not apply any hard thresholds like a plane *P*-value cutoff, which may determine the loss of important biological information. To build a gene network and detect modules, we used the “blockwise” method that is designed for a large dataset; it applies a two-steps clustering, first pre-clusters genes into smaller blocks that include not more than 2,000 genes and then performs a full network analysis in each block separately. Eventually, for each block, modules of genes with high similarity are merged. The results of this analysis are represented by a clustering dendrogram of genes, where each module is represented by a different color.

To relate modules to our study phenotypes, we used a gene-set analysis named ROAST which stands for “rotation gene set testing”(26); this method is implemented as a function in the Limma package (27). Specifically, we tested genes within each module for differential expression across conditions (CPH, SIH, and naïve) and time (week 1 and week 7) using a linear model; all statistics were computed as z-scores and multiple test correction was performed using Benjamini-Hochberg adjustment. ROAST implements different statistics depending on the target proportion of differentially expressed within the gene set. In this study, we used the ‘mean of squared gene-wise statistic' as it is the most indicated in the case where a smaller proportion of genes within the set are differentially expressed. We did not consider the gene-sets or modules with a proportion of DEGs < 30%.

### Enrichment Analysis

We used the web-based dataset Metascape (28) and the R package clusterProfiler (29, 30) to analyze both DEGs and WGCNA modules gene-sets for enrichment in biological process and pathway enrichment, respectively. In this study, we mainly used Gene Ontology (GO) (31), Kyoto Encyclopedia of Genes and Genomes (KEGG) (32), and Reactome (33).

## Results

### Samples

At week 1 after the induction of stress, 103 RNA samples were extracted from the blood, colon, spinal, and DRG of 13 female rats randomly divided into three groups: naïve (*n* = 4), SIH (*n* = 5), and CPH (*n* = 4). Summary statistics of sequences alignment showed that on average 92.2% of the reads mapped properly to the reference, 77.5% to exons, 10% to introns, and 12.4% to intergenic regions (Supplementary Table 1A). An additional 11 female rats were randomly divided into naïve (*n* = 3), SIH (*n* = 4), and CPH (*n* = 4) groups, and 7 weeks after stress induction, RNA was extracted from blood, colon, spinal, and DRG, collecting a total of 72 RNA samples. Alignment summary statistics showed that on average 95.6% of the sequence reads mapped properly to the reference, 77.8% mapped to exons, 9.4% to introns, and 12.8% to intergenic regions of the genome (Supplementary Table 1B). Table 1 summarizes the samples utilized in this study, overall and by condition, time, and tissue.

**Table 1:** Samples stratified by condition, tissue, and time elapsed since stress induction.

	**Week 1**				**Week 7**				
**Condition**	**Blood**	**Colon**	**DRG**	**Spinal**	**Blood**	**Colon**	**DRG**	**Spinal**	**Total**
Naïve	4	15	5	15	4	7	4	9	63
SIH	4	12	4	12	2	6	3	9	52
CPH	4	12	4	12	4	8	4	12	60
Total	12	39	13	39	10	21	11	30	175

Because two different Illumina platforms were used to sequence samples at week 1 and week 7, we had to correct for known batch effect and this step determined a remarkable reduction in the number of genes used for downstream analyses, particularly for blood samples, as 47% of the original genes were excluded.

### Differential Gene Expression Analysis

#### Blood

##### Week 1

One week after stress induction no gene showed a significant difference in expression between CPH and SIH and naive rats, while 17 genes were downregulated in CPH compared to SIH rats (Supplementary Table 2A).

##### Week 7

Seven weeks after stress induction, we observed several DEGs, mainly upregulated in SIH and CPH compared to naïve rats (Table 2 and Supplementary Tables 2B,C).

**Table 2:** Differentially expressed genes across conditions and time elapsed from stress induction across all tissues.

**Tissue**		**Week 1**			**Week 7**		
		**Total significant DE genes (FDR ≤0.05)**	**Upregulated**	**Downregulated**	**Total significant DE genes (FDR ≤0.05)**	**Upregulated**	**Downregulated**
Blood	SIH_*vs*._Naive	0	0	0	23	20	3
	CPH_*vs*._Naive	0	0	0	44	39	5
	CPH_*vs*._SIH	17	0	17	3	1	2
Colon	SIH_*vs*._Naive	23	12	11	2,783	1,342	1,441
	CPH_*vs*._Naive	433	202	231	901	348	553
	CPH_*vs*._SIH	94	46	48	157	62	95
Spinal	SIH_*vs*._Naive	1352	595	757	487	226	261
	CPH_*vs*._Naive	2,630	1,329	1,301	3		3
	CPH_*vs*._SIH	37	7	30	451	214	237
DRG	SIH_*vs._*Naive	0	0	0	0	0	0
	CPH_*vs*._Naive	0	0	0	1	1	0
	CPH_*vs*._SIH	0	0	0	0	0	0

Twenty-one of 23 DEGs in the comparison between SIH and naïve rats, were upregulated in stressed rats and showed significant enrichment in both GO and Reactome “translation” term (adj *P* = 2.3e-03) (Supplementary Figure 1 and Supplementary Tables 2B, 3). The most highly expressed gene in SIH rats was *Ncs1* [fold change (FC) = 32.23], a member of neuronal calcium sensor proteins active in synaptic transmission and plasticity that has been previously found upregulated under stressful conditions (34). Another highly expressed gene in SIH rats was *Hells* (helicase, lymphoid-specific) (FC = 13.38) involved in DNA repair.

Forty-four genes were DE between CPH and naïve rats at week 7, and 39 of them were upregulated in CPH rats (Supplementary Table 2C). These genes mostly encoded for ribosomal proteins and showed significant enrichment in the ribosome metabolism (adj *P* = 8e-23) and oxidative phosphorylation (*P* = 2.3e-05) terms (Supplementary Figure 2 and Supplementary Table 3).

Only three genes (*Dhrsx, Nkap, Top2a*) were differentially expressed between CPH and SIH rats in blood at week 7 (Supplementary Table 2D).

#### Colon

##### Week 1

Twenty-three genes were differentially expressed between SIH and naïve at week 1 in the colon (Table 2 and Supplementary Table 4A); the gene with the highest fold-change, *Aqp8* (FC = 2.67) is a member of a family of water-specific, membrane-channel proteins with a critical role in the nervous system homeostasis and neuronal signaling (35).

Over 400 genes showed differential expression in CPH and naïve rats at week 1 (Table 2 and Supplementary Table 4B). Downregulated genes in CPH rats showed highly significant enrichment in the RNA metabolism (adj *P* ≤ 1.1e-14) and oxidative phosphorylation GO terms (adj *P* ≤ 3.7e-10) (Supplementary Figure 3 and Supplementary Table 3).

Ninety-four genes were DE between CPH and SIH at week 1 (Table 2 and Supplementary Table 4C). Three genes showed a FC ≥ 3 in CPH compared to naïve rats: *Cyp2b1, Neurog3*, and *Nlrp2. Cyp2b1*, a member of the hepatic cytochrome P450 enzyme family, has been described in the initiation and persistence of the pathologic pain by directly activating sensory neurons and inflammatory cytokines (36). *Neurog3* is highly expressed in endocrine progenitor cells and is regulated by the neurotrophic tyrosine kinase receptor type 1 (TRKB-T1), a key regulator of neuronal cell survival, and differentiation. Finally, mRNA levels of *Nlrp2* were previously found to be significantly increased in the DRG of an inflammation-induced pain hypersensitivity mice model (37).

##### Week 7

We observed ~ 2,800 DEGs between SIH and naïve rats (Table 2). Upregulated genes in SIH rats were significantly enriched in several GO terms relevant to histone and chromatin modification, and embryonic and cell morphogenesis (Supplementary Figure 4A and Supplementary Table 3). Genes downregulated in SIH rats were strongly enriched in cellular response to stress (adj *P* = 5.4e-17) and in the RNA metabolism Reactome terms (adj *P* = 2.3e-16) (Supplementary Figure 4B and Supplementary Table 3).

Approximately 3% of DEGs showed a ≥ 2-fold higher expression in SIH compared to naïve rats (Supplementary Table 4D). The top upregulated gene in SIH rats was *Cyp1a1* (FC = 5.41), a member of the hepatic cytochrome P450 enzyme family involved in the metabolism of xenobiotics. Another gene upregulated in SIH rats was *Shank2* (FC = 2.35) a member of scaffold proteins in the postsynaptic density (PSD) of the glutamatergic synapses. *Shank2* knockdown showed a reduction in the response of active synapses (38).

We identified ~ 900 DEGs between CPH and naïve rats at week 7 and 77% of them overlapped with DEGs detected in SIH *vs*. naïve analysis (Supplementary Table 4E). Similar to stressed rats, upregulated genes in CPH rats were significantly represented in chromatin modification (adj *P* = 4.2e-05) and embryonic morphogenesis (adj *P* = 2.2e-02) terms (Supplementary Figure 5A and Supplementary Table 3), while downregulated genes were involved in the regulation of the hypoxia-inducible factor HIF abundance and oxygen homeostasis (adj *P* = 2.9e-10) and innate immune system (adj *P* ≤ 5.5e-07) Reactome terms (Supplementary Figure 5B and Supplementary Table 3).

A total of 157 genes were differentially expressed between CPH and SIH rats at week 7 (Table 2 and Supplementary Table 4F); genes upregulated in CPH were enriched in peptidyl-proline modification GO process (adj *P* = 1.5e-02), while downregulated genes were represented in histone methylation (adj *P* = 3.1e-03) and circadian rhythm (adj *P* ≤ 5.6e-02) (Supplementary Figures 6A,B and Supplementary Table 3). Interestingly, *Lyc2* alias *Lyz2* was the top upregulated gene in CPH compared to SIH rats (FC = 14.01) and it has been recently found associated with nerve injury-induced neuropathic pain (39). As to genes downregulated in CPH compared to SIH rats, they showed downregulation of *Fut9* (FC = −4.20) a gene involved in the synthesis of the Lewis motif; decreased levels of this protein have been associated with a reduction in neurite formation and outgrowth (40).

#### Spinal

##### Week 1

After 1 week of stress induction, we observed 1,352 DEGs between SIH and naïve rats (Table 2 and Supplementary Table 5A); the genes found up-regulated in stressed rats were enriched in the GO response to insulin stimulus biological process (adj *P* = 7e-02) (Supplementary Figure 7A and Supplementary Table 3), while those downregulated were enriched in protein modification GO terms (adj *P* = 1.8e-06) (Supplementary Figure 7B). Two olfactory receptors*, Olr35* and *Olr63*, were 2-fold more expressed in SIH rats compared to naïve. Olfactory perception is affected in stressed animals, as glucocorticoids might enhance odor detection, starting at the first step of detection (41).

We found 2,630 DEGs between CPH and naive rats (Table 2 and Supplementary Table 5B); upregulated genes were enriched in vascular development (adj *P* = 7.4e-04) (Supplementary Figure 8A and Supplementary Table 3), while downregulated genes were significantly represented in RNAs metabolism (adj *P* = 2.6e-30) (Supplementary Figure 8B, Supplementary Table 3). *Capns1* was the most upregulated gene in the comparison of CPH and naïve rats. *Capns1* is a member of a calcium-activated protease family abundant in the CNS and calpain-1 loss leads to reduced dendritic complexity and spine density deficits associated with major deterioration in hippocampal long-term potentiation and spatial memory (42). *Fut9* (FC = −4.91) was the top downregulated gene in CPH compared to naive rats at week 1.

Thirty-seven genes were differentially expressed between CPH and SIH rats (Table 2 and Supplementary Table 5C) and they were significantly enriched in the translation GO biological process (adj *P* = 5.37E-06) (Supplementary Figure 9 and Supplementary Table 3).

##### Week 7

We observed 487 DEGs between SIH and naïve rats; the upregulated genes in stressed rats were significantly enriched in the cell morphogenesis (adj *P* =1.7e-04) and histone modification (adj *P* = 7.1e-04) (Supplementary Figure 10A and Supplementary Table3) and *Atp1a4*, a sodium/potassium-transporting ATPase, was the most upregulated gene in SIH compared to naïve rats (FC = 3.25) (Table 2 and Supplementary Table 5D). Genes found downregulated in stressed rats compared to naïve were significantly enriched in the RNA metabolism (adj *P* =1.7e-10) (Supplementary Figure 10B and Supplementary Table 3).

Three genes (*Ubd, Ciart*, and *Eral1*) showed significantly lower expression in CPH compared to naïve rats at week 7 (Table 2 and Supplementary Table 5E) and ubiquitin D was the most downregulated gene in CPH rats. Interestingly, activation in the ubiquitin-proteasome system (UPS) targets and degrades proteins critical for the maintenance of chronic pain as those involved in synaptic plasticity (43, 44).

We observed 451 DEGs between CPH and SIH conditions (Table 2 and Supplementary Table 5F). The top upregulated genes in CPH compared to SIH rats were *Cyp2d3*, a member of the p450 xenobiotic-inducible superfamily, and lysozyme 2 (*Lyc2*), the enzyme that was also the top upregulated gene in CPH compared to SIH rats at week 7 in the colon. Overall, upregulated genes in CPH rats were enriched in the metabolism of RNA and proteins terms (adj *P* = 1.3e-05)

(Supplementary Figure 11A and Supplementary Table 3), while downregulated genes were represented in tube morphogenesis (adj *P*-value = 4e-03) (Supplementary Figure 11B and Supplementary Table 3).

#### DRG

##### Week 1 and Week 7

We did not observe any DEGs at week 1, whereas at week 7 only AABR07054370.1 alias *Chmp4b* was found significantly upregulated (FC = 1.72, *P*-value = 2.7e-07, FDR = 4.5e-03) in CPH compared to naïve rats. An in vitro study reported that *Chmp4b* may play a role in neuronal apoptosis and could be related to brain damage following intracerebral hemorrhage (45).

### WGCNA-ROAST Analysis

#### Blood

**Figure 1:**
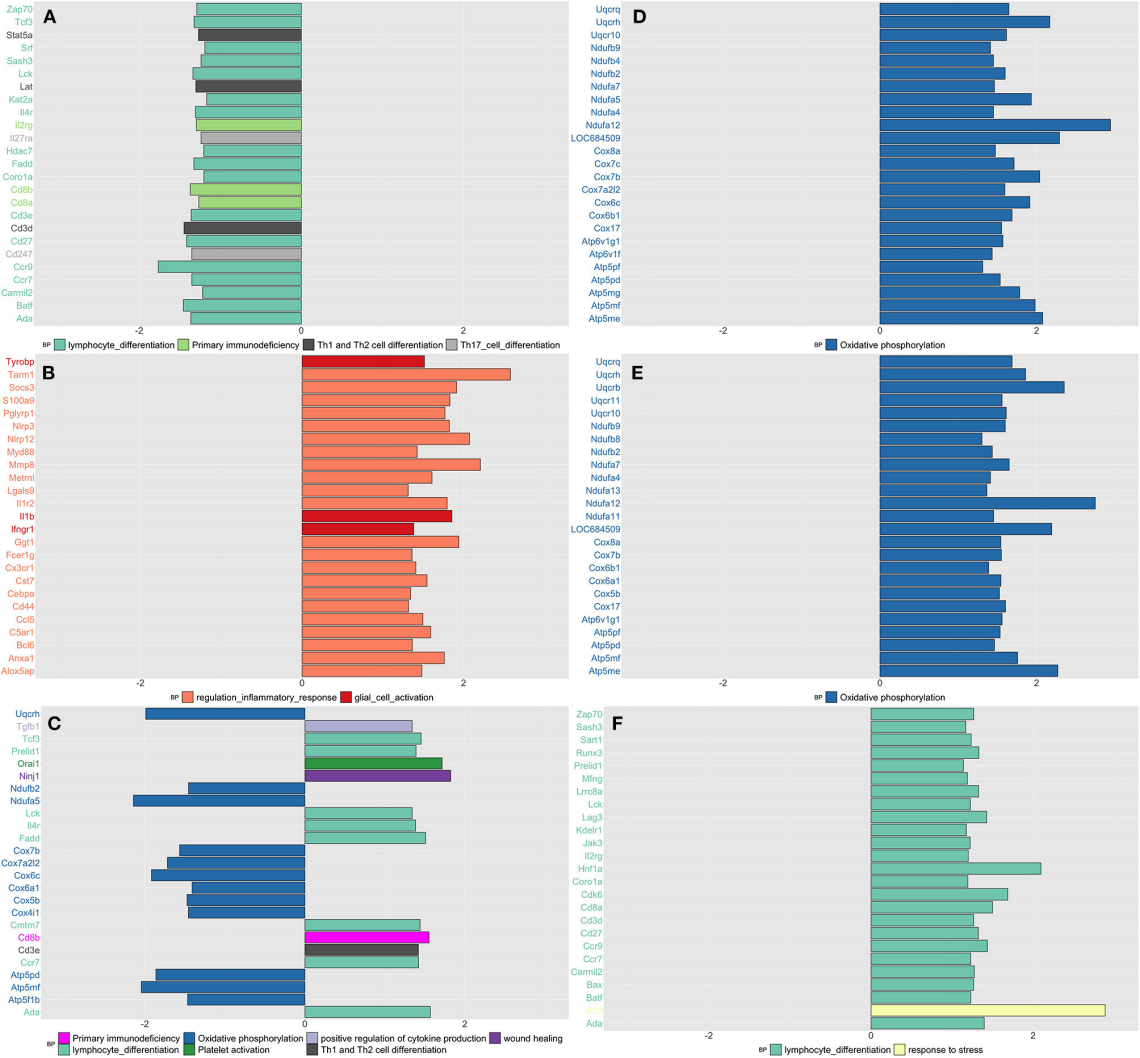
Summarizes the results of both gene-set and gene differentially expressed (DE) in blood tissue across conditions and time. We focused on the top DE gene-sets and top 25 most significant DEGs that were enriched mostly in inflammatory and neurological pathways. **(A)** SIH vs. Naïve at week 1, **(B)** CPH vs. Naïve at week 1, **(C)** CPH vs. SIH at week 1, **(D)** SIH vs. Naïve at week 7, **(E)** CPH vs. Naïve at week 7, and **(F)** CPH vs. SIH at week 7.

By applying network analysis, the entire blood transcriptomic profile was divided into 30 modules of genes grouped by similarity of their eigengene expression profiles (Supplementary Figure 12). Out of the 30 modules, 11 showed at least 30% of their genes up or downregulated in CPH or SIH rats; for each one of these modules, we showed the top three most significant GO biological processes and/or KEGG pathways, prioritizing those related with inflammatory and neurological functions (Supplementary Table 6). As shown in Figure 1, we schematize the results of this analysis and display the top 25 most significant DEGs for each contrast and time point.

##### Week 1

We observed a decreased expression of genes enriched in inflammatory biological processes in stressed rats compared to naïve (Figure 1A), as opposed to stressed CPH rats that compared to the naive ones, showed a higher expression of genes with similar inflammatory functions (Figure 1B). Therefore, CPH rats compared to stressed-only rats showed activation of inflammatory and wound healing processes accompanied by a decreased expression of genes involved in the metabolic pathway of oxidative phosphorylation (Figure 1C).

##### Week 7

SIH and CPH rats showed higher expression of genes enriched in oxidative phosphorylation when compared to naïve rats (Figures 1D,E). Similarly, to what we observed at week 1, at week 7 CPH rats compared to SIH showed activation of inflammatory and stress response biological processes (Figure 1F).

#### Colon

The entire gene expression profile extracted from the colon clustered into 40 modules (Supplementary Figure 13) and 26 of them were significantly associated with CPH or SIH conditions at week 1 or week 7 from stress induction (Supplementary Table 7).

##### Week 1

**Figure 2:**
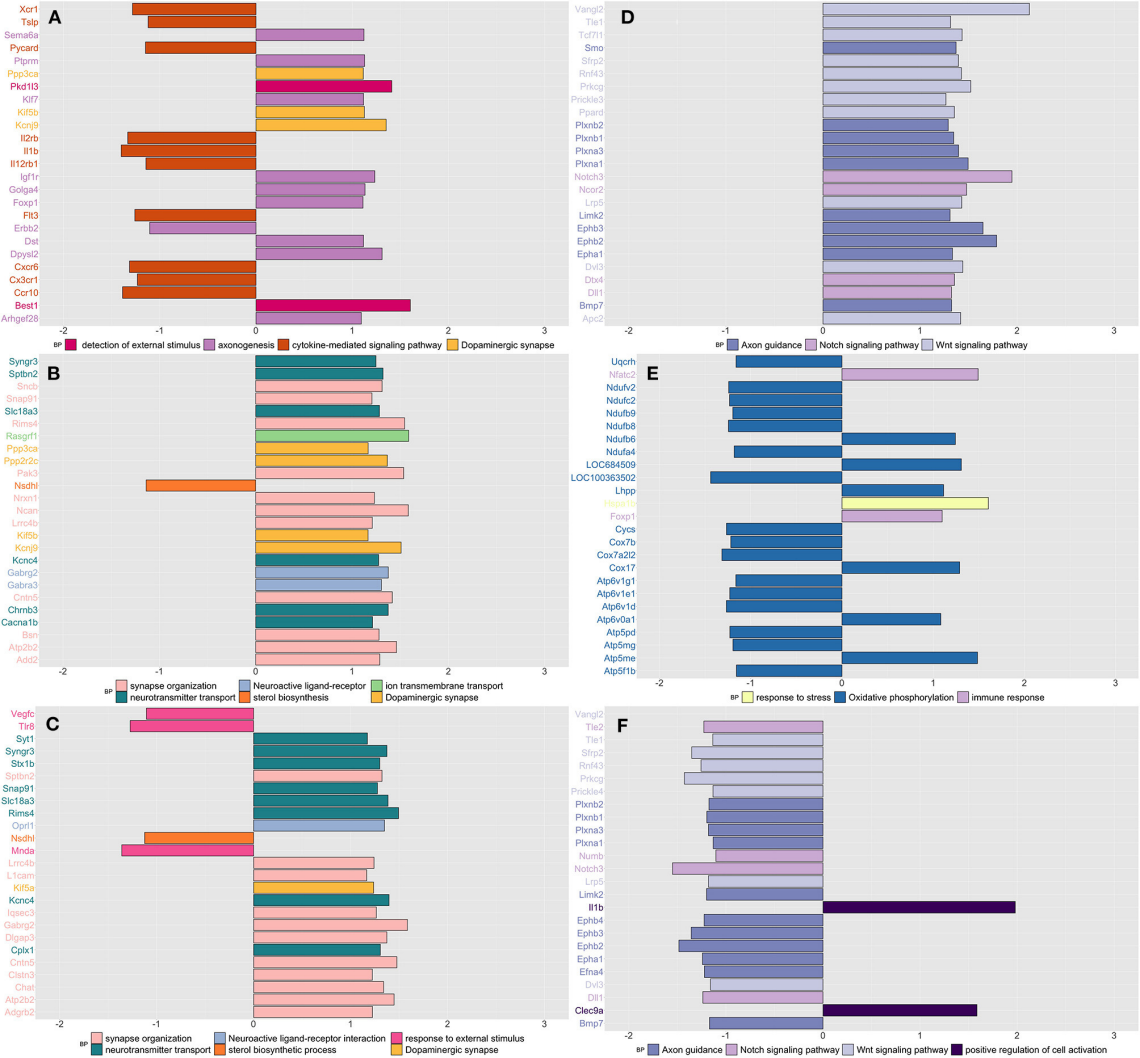
Summarizes the results of both gene-set and gene differentially expressed (DE) in colon tissue across conditions and time. We focused on the top DE gene-sets and top 25 most significant DEGs that were enriched mostly in inflammatory and neurological pathways. **(A)** SIH vs. Naïve at week 1, **(B)** CPH vs. Naïve at week 1, **(C)** CPH vs. SIH at week 1, **(D)** SIH vs. Naïve at week 7, **(E)** CPH vs. Naïve at week 7, and **(F)** CPH vs. SIH at week 7.

Similar to what we have observed in blood, stressed rats showed downregulation of genes enriched in inflammation (cytokine-mediated), as opposed to an increased expression of genes enriched in nervous system processes such as detection of external stimulus, axonogenesis, and dopaminergic synapsis (Figure 2A and Supplementary Table 7). CPH rats compared to both naïve and SIH ones, showed higher expression of genes involved in neuronal functions, mostly related with synapses (Figures 2B,C and Supplementary Table 7). In addition, we observed that genes involved in the response to an external stimulus were downregulated in CPH compared to SIH rats (Figure 2C and Supplementary Table 7).

##### Week 7

Stressed rats compared to CPH and naïve, showed upregulation of genes enriched in axon guidance, Notch, and Wnt signaling pathways (Figures 2D,F and Supplementary Table 7). CPH rats compared to naive showed upregulation of immune response and response to stress genes, while genes enriched in oxidative phosphorylation were mostly downregulated (Figure 2E and Supplementary Table 7).

#### Spinal

Network analysis applied to the whole spinal transcriptomic profile identified 37 modules (Supplementary Figure 14) and 27 of them were found significantly up or downregulated in CPH and/or SIH conditions at week 1 and/or week 7 from stress induction (Supplementary Table 7).

##### Week 1

**Figure 3:**
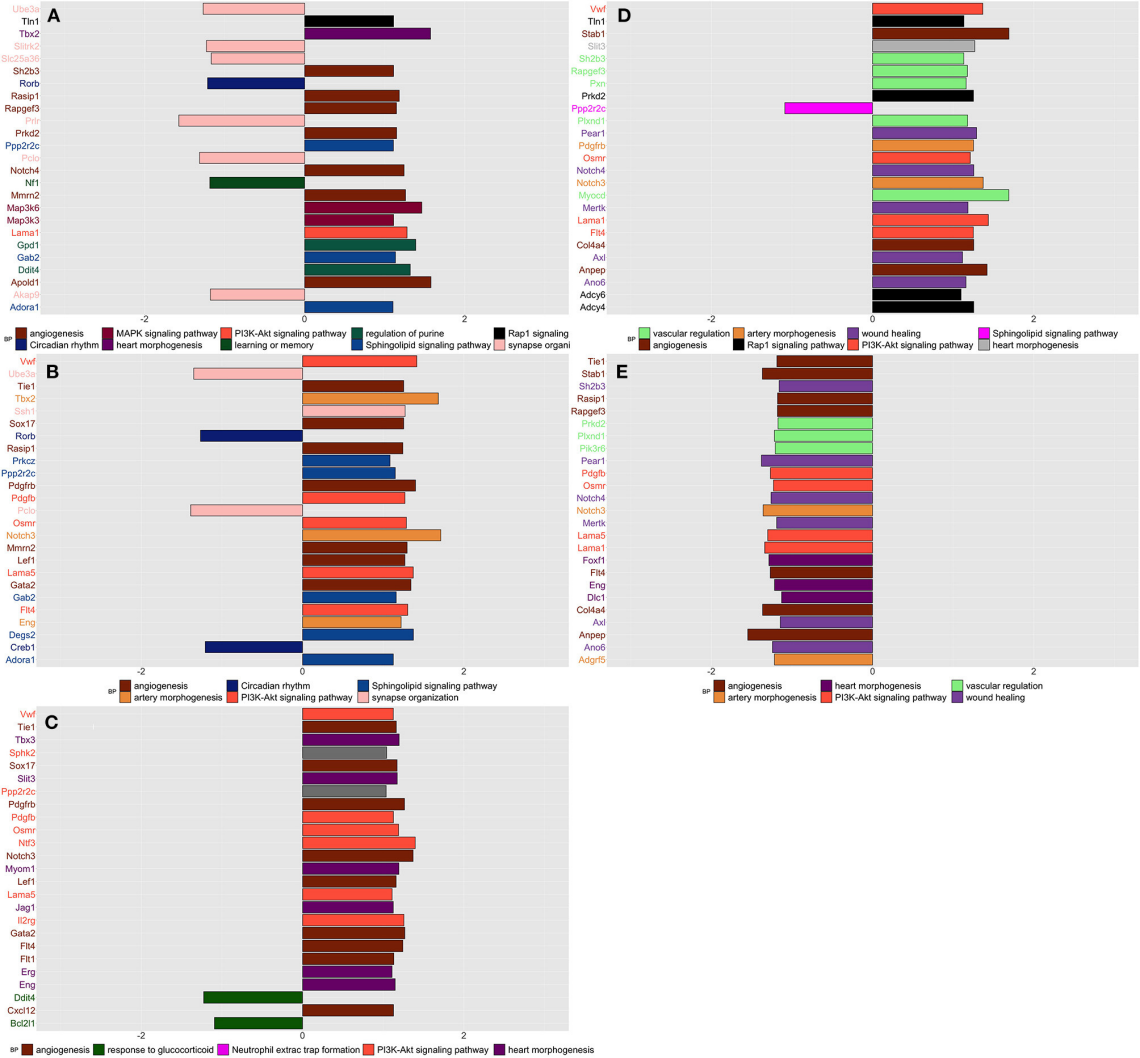
Summarizes the results of both gene-set and gene differentially expressed (DE) in spinal tissue across conditions and time. We focused on the top DE gene-sets and top 25 most significant DEGs that were enriched mostly in inflammatory and neurological pathways. **(A)** SIH vs. Naïve at week 1, **(B)** CPH vs. Naïve at week 1, **(C)** CPH vs. SIH at week 1, **(D)** SIH vs. Naïve at week 7, **(E)** CPH vs. Naïve at week 7.

Compared to naive, SIH and CPH rats showed decreased expression of genes linked to the organization of synapses and circadian rhythm biological processes together with an increased expression of genes related to different biological processes and pathways finalized to the production of new blood vessels, such as “angiogenesis” and “phosphatidylinositol 3-kinase (PI3K) “(Figures 3A,B). Compared to SIH, CPH rats showed an increased expression of genes associated with the vascularization process along with a decreased expression of genes linked to response to glucocorticoids (Figure 3C).

##### Week 7

At week 7 after the induction of stress, we observed a reversal of the trend in the vascularization process; genes linked to the creation of new blood vessels as “angiogenesis” “phosphatidylinositol 3-kinase and wound healing showed higher expression in stressed compared to comorbid rats (Figures 3D,E)

#### DRG

**Figure 4:**
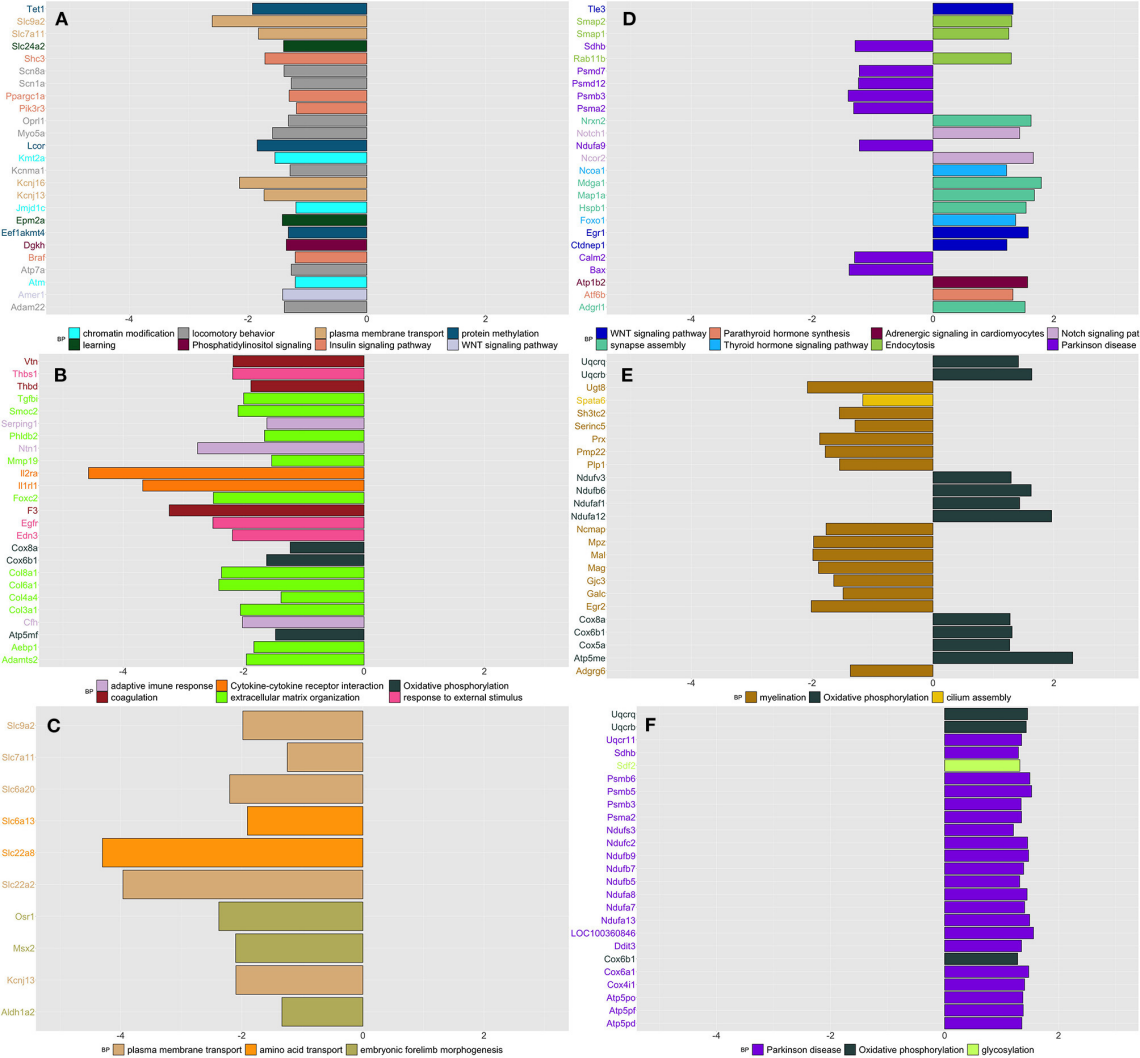
Summarizes the results of both gene-set and gene differentially expressed (DE) in DRG tissue across conditions and time. We focused on the top DE gene-sets and top 25 most significant DEGs that were enriched mostly in inflammatory and neurological pathways. **(A)** SIH vs. Naïve at week 1, **(B)** CPH vs. Naïve at week 1, **(C)** CPH vs. SIH at week 1, **(D)** SIH vs. Naïve at week 7, **(E)** CPH vs. Naïve at week 7, and **(F)** CPH vs. SIH at week 7.

WGCNA network analysis applied to the whole DRG transcriptomic profile divided the genes into 50 modules (Supplementary Figure 15); 20 of these modules showed ≥ 30% of their genes significantly upregulated in at least one condition and time point (Supplementary Table 8). Figure 4 shows the core enrichment of the top 25 DEGs in the related GO and KEGG terms.

##### Week 1

We observed that a significant proportion of genes clustering in different modules were downregulated in SIH compared to naïve rats. These genes were enriched in “chromatin modification,” “Insulin signaling pathway,” “learning,” “locomotory behavior,” “Phosphatidylinositol signaling,” “plasma membrane transport,” “protein methylation,” and “Wnt signaling pathway” (Figure 4A and Supplementary Table 8).

Likewise, in the contrast between CPH and naïve rats at week 1, we found that a significant proportion of genes clustering within different modules were downregulated in CPH rats (Supplementary Table 8). These genes were enriched in inflammatory processes such as “adaptive immune response,” “cytokine–cytokine receptor interaction,” and “complement and coagulation cascades.” (Figure 4B).

When we compared directly CPH to SIH rats at week 1, we found that all genes were downregulated in CPH rats and the highest number of genes were enriched in “plasma membrane transport,” followed by “embryonic forelimb morphogenesis” and “amino acid transport” (Figure 4C).

##### Week 7

We observed different enrichment patterns for all contrasts compared to those observed in week 1. SIH rats compared to naïve showed upregulation of genes enriched in neurological processes as “response to axon injury” and “synapse organization. Conversely, genes downregulated in SIH rats were overrepresented in neurodegenerative diseases and the processing of different RNAs (Figure 4D and Supplementary Table 8).

CPH rats compared to naive showed higher expression of genes enriched in oxidative phosphorylation and downregulation of genes involved in the nerve myelination (Figure 4E).

When compared to SIH rats, CPH showed upregulation of genes involved in the oxidative phosphorylation GO biological process and Parkinson's disease KEGG pathway (Figure 4F).

### Identification of CPH Biomarkers

**Figure 5:**
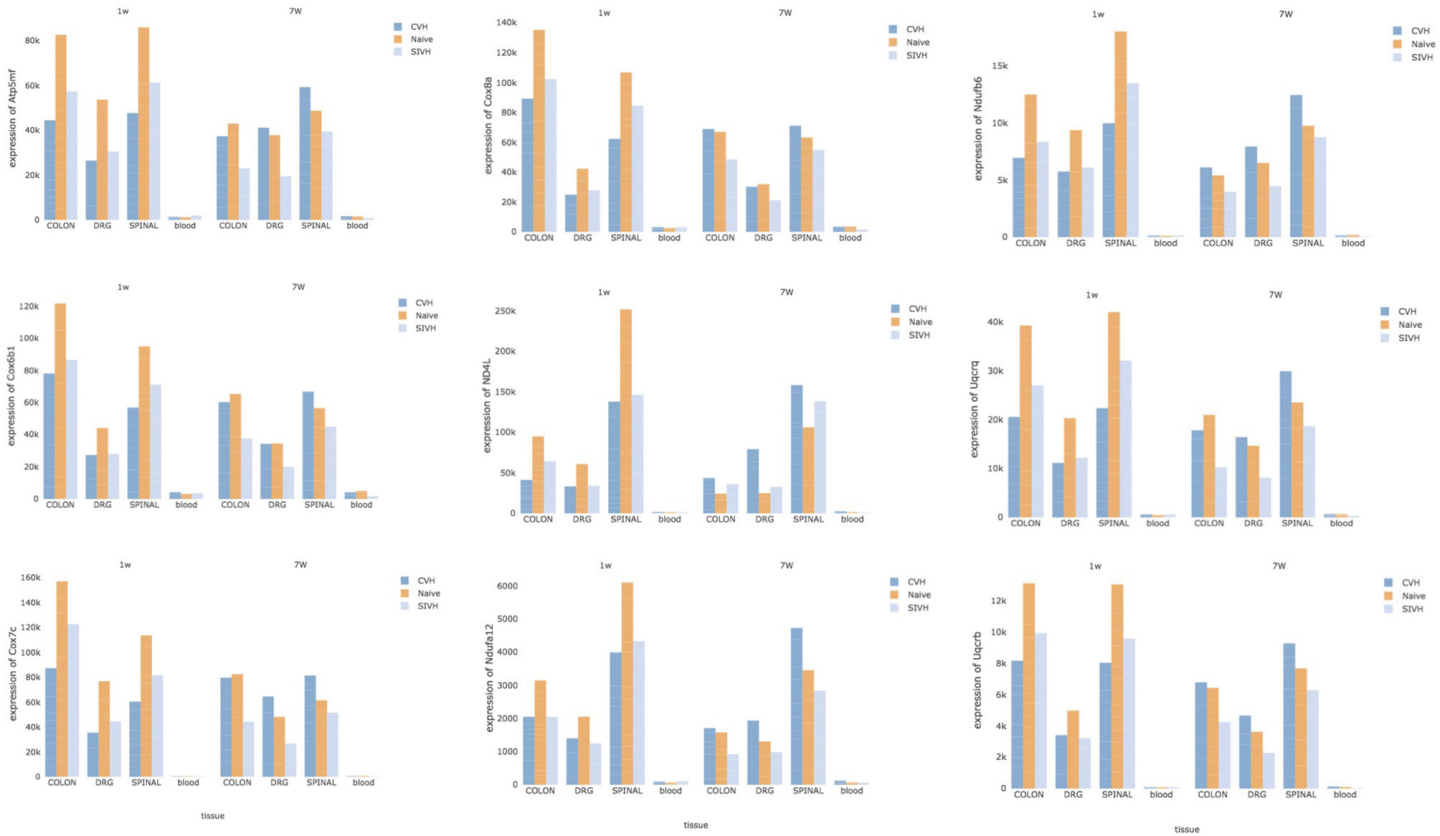
Potential CPH biomarkers in blood. The figure shows 9 genes, representative of mitochondrial membrane ATP synthase, cytochrome c oxidase, NADH: ubiquinone oxidoreductase (complex I) and ubiquinol-cytochrome c oxidoreductase complex. Since these genes showed high correlation of their expression profiles across the solid tissues (DRG, colon, and spinal) and blood, they represent potential diagnostic biomarkers for CPH.

Finally, to detect specific CPH biomarkers from blood, we focused our attention on the genes or gene-sets that are distinctive of the comorbid condition and looked for any correlation of their expression profiles across the solid tissues (DRG, colon, and spinal) and blood. Both DE at the gene and gene-set levels pointed to distinct mitochondrial genes: members of the mitochondrial membrane ATP synthase (*Atp5me, Atp5mf* , and *Atp5pd*), representatives of the cytochrome c oxidase (*Cox6b1, Cox7c*, and *Cox8a*), members of the mitochondrial NADH: ubiquinone oxidoreductase (complex I) (*Nd4l, Ndufa12*, and *Ndufb6*) and components of the ubiquinol-cytochrome c oxidoreductase complex (*Uqcrb, Uqcrh*, and *Uqcrq*). Despite the low abundance of these genes in blood compared to the other tissues, we noticed a common trend in the expression of these genes across all tissues, conditions, and time points (Figure 5). At week 1, CPH rats showed the lowest expression of these genes compared to SIH and naïve, while at week 7 we observed a complete inversion of this trend, with the higher expression in comorbid rats followed by naïve and SIH rats (Figure 5).

The observed correlation in the expression of these genes reflected a highly significant functional interaction of their proteins; in fact, Search Tool for the Retrieval of Interacting Genes/Proteins detected 66 protein-protein interactions compared to the expected 5 (PPI, *P* ≤ 1.0e-16) (Supplementary Figure 16) and reported high enrichment in the oxidative phosphorylation biological process (adj *P* = 1.1e-09).

## Discussion

Several studies have shown a correlation between stress and pain and that chronic exposure to stress increases the intensity of the pain (1, 2). Stress is a major underlying factor in IBS (1, 6, 9, 10) and TMD (46, 47), another chronic pain condition, and these conditions are significantly comorbid (11, 12). In this study, we explored if the genetic mechanisms that are triggered in the development of pain due to stress are the same as those evoked by the presence of multiple comorbid pain conditions. To answer this question, we compared the entire transcriptomic profile of CPH rats to both SIH and control non-stressed rats and focused on two major biological components involved in pain: inflammation and the nervous system.

We carried out the analysis in four different tissues: at the systemic level in the blood, and in the colon, DRG, and spinal tissues. Blood was studied because, given the complexity of overlapping pain conditions, easily available biomarkers may provide the early identification and diagnosis of these disorders and eventually be applied to translational research on chronic visceral pain among human subjects. Colon tissue is commonly used in transcriptomic analyses applied to humans affected with IBS and animal models, as it may pave the way for the gastrointestinal origin of stress-induced chronic visceral pain (48, 49). The spinal cord and dorsal root ganglia are key stations in sensory transduction and modulation, including pain transmission (50).

The analysis of the whole transcriptome profile in blood and colon tissues at week 1 showed activation of the inflammatory response in CPH compared to SIH rats and in the colon, this effect persisted after 7 weeks from stress induction. Previous studies described a long-term decrease in the number of T cells in peripheral blood after repeated chronic mild stress induction (51, 52). Furthermore, in blood and colon tissues at week 1, CPH rats compared to SIH showed increased expression of genes enriched in synapse organization, and neurotransmitter transport biological processes. In the colon, at week 7 we observed significantly higher expression of genes enriched in Wnt and Notch pathways in SIH compared to CPH rats. The wnt/β-Catenin pathway has many critical regulatory functions, including those related to the axis formation and synapse development and modulation (53). A recent report shows that the Wnt/β-Catenin pathway boost ribosome biogenesis in response to stress, allowing cells to grow and survive (54). One week after stress induction, the transcriptional profile from spinal tissue showed a similar picture in SIH and CPH rats; the two compared to naïve showed a decreased expression of genes linked to the synaptic organization, likely because of the damage in the nerve tissue due to stress exposure. Simultaneously, the exposure to stress in these rats, particularly in CPH rats, determined an increased expression of genes enriched in biological processes and pathways aimed at the production of new blood vessels, angiogenesis, and phosphatidylinositol 3-kinase (PI3K), and neurotrophin pathways. The PI3K pathway plays an important role in regulating angiogenesis; hypoxia leads to the hypoxia-inducible factor 1-alpha (HIF-1α) stabilization and is a major stimulus for increased vascular endothelial growth factor (VEGF) production (55).

The PI3K /Akt pathway has been reported as a regulator of spinal plasticity in the rat visceral pain model (56) and associated with chronic pain (57). Previous studies described an increased production of VEGF by intestinal mucosa cells of patients with IBS and associated vascular endothelial growth factors with visceral hyperalgesia, abdominal discomfort, and/or pelvic pain (58, 59). Likewise, the neurotrophin pathway promotes blood vessel growth (60); neurotrophins such as NT-3 control the sympathetic innervation of blood vessels (60, 61). Blood vessels play a crucial role in nerve regeneration; in physiological conditions, they transport ingredients (oxygen, nutrients, and hormones), remove metabolic waste, and facilitate cell circulation, which provides a supportive microenvironment for the nervous system (62). This mechanism may contribute to the recovery from nervous damage by stress induction. Interestingly, our study highlights that the representative genes to the angiogenesis-related terms differed between CPH and SIH rats. Furthermore, angiogenesis-related genes were more highly expressed in CPH than SIH rats 1 week after stress, while the situation was reversed 7 weeks after stress exposure.

Genes related to “response to glucocorticoid” were downregulated in the CPH compared to SIH rats, suggesting negative feedback in response to the stronger stress due to persistent sensitization through 7 weeks (13). In addition, at 7 weeks from stress induction, “wound healing” was also downregulated in CPH in comparison to SIH rats; it is well known that stress and pain can deteriorate wound healing (63).

Our results suggest that chronic stressful condition in the CPH rats contributes to the delayed healing from tissue damage in the nervous system, resulting in the prolonged referred pain observed in our previous study (13).

Finally, we analyzed the transcriptome profile obtained from DRG at week 1 and week 7 after stress induction. At week 1, all the inflammatory and neurological response-related genes were downregulated in both SIH and CPH rats compared to naive. However, at week 7, SIH rats compared to naïve showed upregulation of genes enriched in the synapse assembly, Wnt and Notch pathways (48) as opposed to CPH rats that revealed a significant proportion of genes enriched in the myelination process were downregulated in CPH compared to naive. However, we did not observe genes enriched in any neurological or inflammatory functions when comparing SIH and CPH at 7 weeks, which is in line with the previous electrophysiological findings that peripheral sensitization was present in both SIH and CPH rats at 7 weeks (13).

Future studies could investigate the long-term effect of stress on pain sensations among CPH rats to determine the impact of the downregulated genes related to the myelination process among these rats. Our results may suggest greater damage in the neurological components following stress in CPH compared to SIH at 7 weeks, although these results are not consistent with behavioral changes.

Our study highlights a common signature across tissues and time elapsed from stress exposure; in all tissues, we noticed that several mitochondrial genes, involved in the oxidative phosphorylation pathway are downregulated in both CPH and SIH compared to naïve rats, with the lower expression in CPH rats at week 1, while at week 7, the same or related genes are upregulated in CPH in comparison to SIH and naïve rats. All biological processes activated by acute or chronic stress require a substantial amount of energy. Mitochondria are the major source of cellular energy, via the transformation of energetic substrates and oxygen into ATP (64). Additionally, all steroid hormones, including progestogens (e.g., progesterone), mineralocorticoids (e.g., aldosterone), glucocorticoids (e.g., cortisol and corticosterone), androgens (e.g., testosterone), and estrogens (e.g., estriol) are synthesized in mitochondria(64). Chronic pain has been associated with oxidative stress. The hypothesized mechanism behind this link is the release of reactive oxygen species, which affects mitochondrial function by reducing the release of ATP in cells (65).

In conclusion, our study highlights time and tissue-specific genetic signatures that help to differentiate the inflammatory and neurological response to stress in SIH and comorbid TMD–IBS pain hypersensitivity (CPH) rats. Moreover, our data support the theory behind the association between pain and oxidative stress, as demonstrated by the decreased expression of mitochondrial genes in SIH and even more conspicuously in CPH rats compared to naïve and reaffirm the value of antioxidants as a therapeutic target for chronic pain. Finally, if we look at the switch in the expression levels of mitochondrial genes between 1 and 7 weeks after stress exposure, and interpret our results in the context of the energy required for cell growth and survival, this study shows that the production of energy for repairing stress-induced damage in SIH rats starts and ends earlier than in CPH rats, where the need of energy to face stress consequences last much longer.

## Data Availability Statement

The data presented in the study are deposited in the NCBI repository (https://www.ncbi.nlm.nih.gov/geo/), accession number GSE199221. Expression data for genes analyzed across all tissues have been uploaded to the Neuroscience Multi-Omic (NeMO) (66) Analytics portal which enables web-based visualization available at the following link (https://nemoanalytics.org/p?s=2f41a962) [accessed February 22, 2022], accessible previous registration.

## Ethics Statement

The animal study was reviewed and approved by University of Maryland Institutional Animal Care and Use Committee.

## Author Contributions

SD and RT contributed to the conception and design of the study. SA contributed to the design of the study. EM performed the statistical analysis. EM, TG, and JC contributed in the manuscript preparation. All authors contributed to manuscript revision and approved the submitted version.

## Funding

This study was funded by the R01 grant: R01NR015472 to Dorsey and Traub.

## Conflict of Interest

The authors declare that the research was conducted in the absence of any commercial or financial relationships that could be construed as a potential conflict of interest.

## Publisher's Note

All claims expressed in this article are solely those of the authors and do not necessarily represent those of their affiliated organizations, or those of the publisher, the editors and the reviewers. Any product that may be evaluated in this article, or claim that may be made by its manufacturer, is not guaranteed or endorsed by the publisher.
