# Supplementary material for: Early and Late Transcriptional Changes in Blood, Neural, and Colon Tissues in Rat Models of Stress-Induced and Comorbid Pain Hypersensitivity Reveal Regulatory Roles in Neurological Disease
Source: Front Pain Res (Lausanne). 2022 May 17;3:886042. doi: 10.3389/fpain.2022.886042 (PMC9152010; doi:10.3389/fpain.2022.886042)
Supplement: Supplementary file 10 [file Data_Sheet_1.docx]

**Suppl. Figure 1.** Enrichment results upregulated genes in SIH VS naïve rats at week7 in Blood ****

**Suppl. Figure 2.** Enrichment results upregulated genes in CPH VS naïve rats at week7 in Blood

**Suppl. Figure 3.** Enrichment results downregulated genes in CPH VS naïve rats at week1 in Colon

**Suppl. Figure4A**. Enrichment results upregulated genes in SIH VS naïve rats at week7 in Colon

**Suppl. Figure4B**. Enrichment results downregulated genes in SIH VS naïve at week7 in Colon

**Suppl. Figure5A**. Enrichment results upregulated genes in CPH VS naïve rats at week7 in Colon

**Suppl. Figure5B**. Enrichment results downregulated genes in CPH VS naïve rats at week7 in Colon

**Suppl. Figure6A**. Enrichment results upregulated genes in CPH VS SIH rats at week7 in Colon

**Suppl. Figure6B**. Enrichment results downregulated genes in CPH VS SIH rats at week7 in Colon

**Suppl. Figure7A**. Enrichment results upregulated genes in SIH VS naïve rats at week1 in Spinal tissue

**Suppl. Figure7B**. Enrichment results downregulated genes in SIH VS naïve rats at week1 in Spinal tissue

**Suppl. Figure8A**. Enrichment results upregulated genes in CPH VS naïve rats at week1 in Spinal tissue

**Suppl. Figure8B**. Enrichment results downregulated genes in CPH VS naïve rats at week1 in Spinal tissue

**Suppl. Figure9**. Enrichment results downregulated genes in CPH VS SIH rats at week1 in Spinal tissue

**Suppl. Figure10A**. Enrichment results upregulated genes in SIH VS naive rats at week7 in Spinal tissue

**Suppl. Figure10B**. Enrichment results downregulated genes in SIH VS naive rats at week7 in Spinal tissue

**Suppl. Figure11A**. Enrichment results upregulated genes in CPH vs SIH rats at week7 in Spinal tissue

**Suppl. Figure11B**. Enrichment results downregulated genes in CPH vs SIH rats at week7 in Spinal tissue

**Supplementary Figure 12.** Network analysis in blood tissue. **A**. Module clusters

**B.** Number of genes clustering into each module

Abbreviations; ME:module

**Supplementary Figure 13.** Network analysis in colon tissue. **A**. Module clusters

**B.** Number of genes clustering into each module

Abbreviations; ME:module

**Supplementary Figure 14.** Network analysis in spinal tissue. **A**. Module clusters

**B.** Number of genes clustering into each module

Abbreviations; ME:module

**Supplementary Figure 15.** Network analysis in DRG tissue. **A**. Module clusters

**B.** Number of genes clustering into each module

**Supplementary Figure 16.** Protein-protein interaction analysis applied to potential CPH biomarker in blood.

**
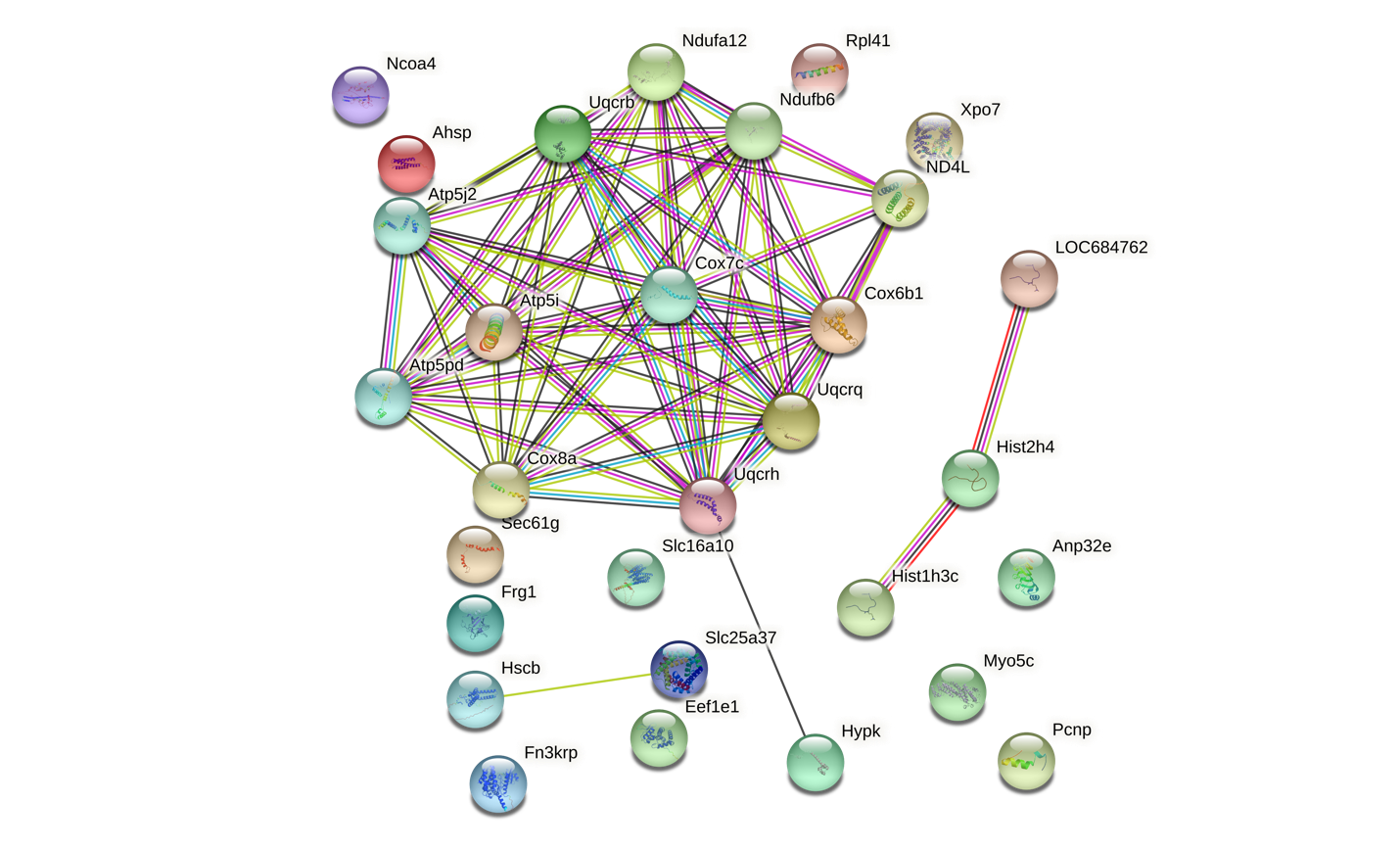
**
